# Distinct Stabilities of the Structurally Homologous Heptameric Co-Chaperonins GroES and gp31

**DOI:** 10.1007/s13361-018-1910-5

**Published:** 2018-05-07

**Authors:** Andrey Dyachenko, Sem Tamara, Albert J. R. Heck

**Affiliations:** 1 Biomolecular Mass Spectrometry and Proteomics, Bijvoet Center for Biomolecular Research and Utrecht Institute for Pharmaceutical Sciences, Utrecht University, Padualaan 8, 3584 CH Utrecht, The Netherlands; 2 Netherlands Proteomics Centre, Padualaan 8, 3584 CH Utrecht, The Netherlands

**Keywords:** GroES, gp31, Chaperone, Native mass spectrometry, Ion mobility mass spectrometry, Gas-phase unfolding

## Abstract

The GroES heptamer is the molecular co-chaperonin that partners with the tetradecamer chaperonin GroEL, which assists in the folding of various nonnative polypeptide chains in *Escherichia coli*. Gp31 is a structural and functional analogue of GroES encoded by the bacteriophage T4, becoming highly expressed in T4-infected *E. coli*, taking over the role of GroES, favoring the folding of bacteriophage proteins. Despite being slightly larger, gp31 is quite homologous to GroES in terms of its tertiary and quaternary structure, as well as in its function and mode of interaction with the chaperonin GroEL. Here, we performed a side-by-side comparison of GroES and gp31 heptamer complexes by (ion mobility) tandem mass spectrometry. Surprisingly, we observed quite distinct fragmentation mechanisms for the GroES and gp31 heptamers, whereby GroES displays a unique and unusual bimodal charge distribution in its released monomers. Not only the gas-phase dissociation but also the gas-phase unfolding of GroES and gp31 were found to be very distinct. We rationalize these observations with the similar discrepancies we observed in the thermal unfolding characteristics and surface contacts within GroES and gp31 in the solution. From our data, we propose a model that explains the observed simultaneous dissociation pathways of GroES and the differences between GroES and gp31 gas-phase dissociation and unfolding. We conclude that, although GroES and gp31 exhibit high homology in tertiary and quaternary structure, they are quite distinct in their solution and gas-phase (un)folding characteristics and stability.

**Electronic supplementary material:**

The online version of this article (10.1007/s13361-018-1910-5) contains supplementary material, which is available to authorized users.

**Figure:**
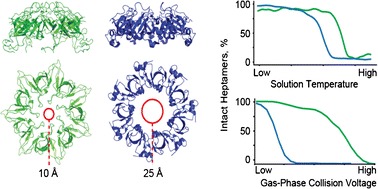
Graphical Abstract

## Introduction

**Figure 1:**
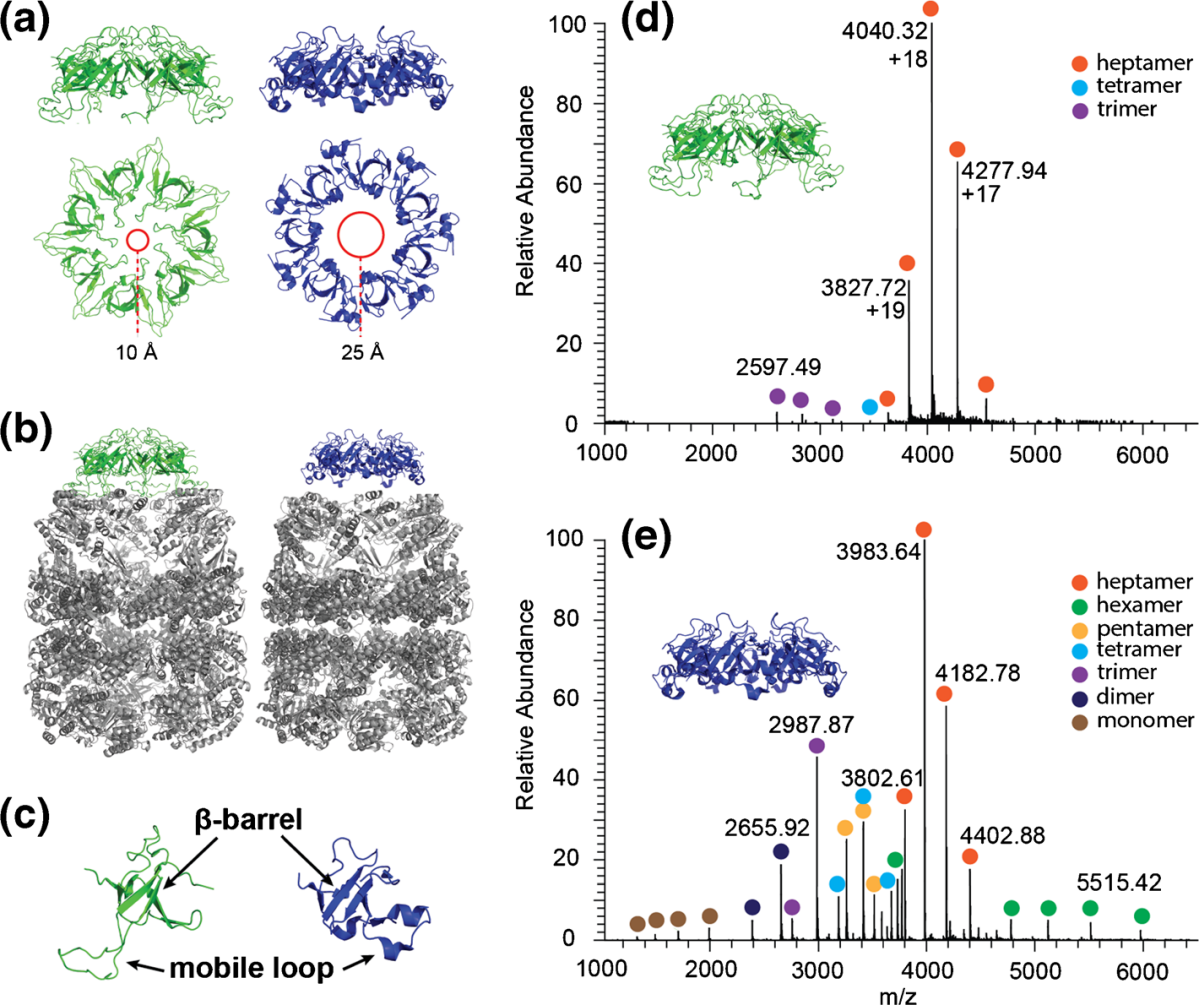
Structural differences and similarities between GroES and gp31. (**a**) Side and top views of the GroES (green) and gp31 (blue) crystal structures (PDB accession codes 1AON and 2CGT, respectively). Red circles highlight the difference in the diameter of the central hole in the gp31 and GroES ring when complexed to GroEL. (**b**) Comparison of GroES and gp31 in their complexes with GroEL. 3D representation of the complexes from the side. (**a**) and (**b**) are adopted from [17]. (**c**) Similarities of GroES and gp31 monomers. (**d**) Native electrospray ionization mass spectra of GroES. (**e**) gp31 revealing the preferential heptameric stoichiometry in aqueous ammonium acetate

The molecular heptameric co-chaperonin GroES forms a cap on top of the *Escherichia coli* molecular tetradecameric GroEL chaperone that assists the folding and refolding of nonnative polypeptide chains (Figure 1). The chaperonin GroEL, GroES, and nonnative protein undergo a *binding* → *folding* → *release* cycle regulated by ATP hydrolysis [1–4]. In this process, GroES acts as a lid that covers the inner cavity of the GroEL chaperonin following the binding of the substrate and ATP inside the Anfinsen cage of GroEL. Formation of the GroEL-GroES complex is only possible when GroEL is in the open conformation (ATP bound). When substrate proteins are absent, GroEL primarily binds one GroES heptamer. In the presence of substrate, however, GroEL could accommodate one or two GroES heptamers, forming either asymmetric bullet-shaped or symmetric US football-shaped complexes, respectively [5]. These complexes are in a folding-active state, providing nano-environment wherein the substrate is free of nonnative interactions that can lead to aggregation [6, 7]. Structurally, GroES is a homo-heptamer with a ring symmetry, constituted of 10.4 kDa monomeric subunits [8] (Figure 1a–c; green). Each subunit comprises a rigid beta-barrel structure and an unstructured loop that forms contacts with the apical domain of a corresponding GroEL subunit upon the GroEL-GroES complex formation [5, 9].

Gp31 is a structural homologue of GroES (Figure 1a–c; blue) encoded by the *E. coli* bacteriophage T4, which is essential for the folding of the T4 major capsid protein gp23 [10, 11]. Gp31 mimics the action of GroES by competitively binding to GroEL and acting as a co-chaperonin. In vitro, the GroEL-gp31 complex is capable of folding substrates such as citrate synthase and Rubisco with an efficiency similar to that of GroEL-GroES [11–13]. In vivo, the GroEL-gp31 complex can substitute the *E. coli* folding machinery and fold all the *E. coli* proteins that normally rely on the GroEL-GroES system [14]. Therefore, gp31 can functionally substitute GroES and additionally can assist in folding of the T4 major capsid protein precursor gp23, which at ~ 56 kDa is slightly larger than the substrates that can be accommodated within the GroEL-GroES Anfinsen cage [15].

Although GroES and gp31 have amino acid sequence identity of only 14%, their tertiary and quaternary structures are similar [16] (Figure 1a–c). Like GroES, gp31 in a solution forms ring-shaped heptamers composed of identical subunits. The fold of the gp31 monomer also consists of a β-barrel and a mobile loop (Figure 1c). There are, however, a few notable differences. The higher molecular weight (MW) of the gp31 monomer (12 versus 10 kDa for the GroES monomer) together with longer flexible loops (22 residues, GroES has 16 residues) result in GroEL-gp31 complexes having a slightly larger internal chamber volume as compared to GroEL-GroES. The direct measurements performed via cryo-electron microscopy (cryo-EM) revealed a size increase of 8%, from 194,000 to 210,000 Å^3^ [17]. Additionally, gp31 does not contain the roof loops that form the top of the dome in the GroEL-GroES complex (Figure 1a), leaving a wider opening and potentially allowing part of the polypeptide substrate chain to stick out. These subtle differences are generally believed to make the GroEL-gp31 complex capable of folding the larger gp23 substrate protein.

Parallel with the development of biomolecular, and especially native, mass spectrometry, there is a continuous debate whether biomolecules retain their native structural properties upon transition into the gas phase [18–20]. Primary arguments against retained structural features in the gas phase point at the role of water and lipid molecules to stabilize and maintain native protein structures [21]. Additionally, in the early days of native MS, researches often observed false positives, i.e., formation of nonspecific complexes upon transfer into the mass spectrometer [22]. However, with improvements in instrumentation (e.g., nano-electrospray ionization), as well as other advances in the fields of native mass spectrometry (native MS) and ion mobility-mass spectrometry (IM-MS), data supporting a more gentle gas-phase transition started to accumulate [23, 24]. Furthermore, nonergodic fragmentation techniques that preferentially preserve noncovalent interactions (e.g., electron-transfer dissociation) revealed similarities between native-like structures of proteins observed in the solution and in the gas phase [25–28]. Taken together, accumulating all the evidence, it has become clear that it is possible to retain partially solution-phase properties of proteins and protein complexes in the gas phase, although it remains system dependent.

GroES and gp31 have been previously examined with gas-phase techniques, both alone [29–31] and in complex with GroEL [32–34]. It has been established that both GroES and gp31 can be maintained in the gas phase as stable heptameric complexes. Here we present a side-by-side comparison of GroES and gp31 gas-phase behavior, revealing substantial differences in their collision induced dissociation (CID) and collision induced unfolding (CIU) behavior. We attribute these differences to the differing structural features of the two complexes in the solution and in the gas phase.

## Materials and Methods

### GroES and gp31

Both co-chaperonins were over-expressed in *E. coli* strain MC1009 [35] and purified as described previously [36]. The aliquots were flash frozen in liquid nitrogen and stored at − 80 °C.

### Thermal Unfolding

For the thermal unfolding assay, the proteins were dissolved in a 50-mM phosphate buffer at pH 6.8 to a final concentration of 0.15 mg/ml. The measurements were taken on a J-810 Spectropolarimeter (Jasco Inc., Easton, MD, USA) using 250 μl cuvette. Both GroES and gp31 were scanned from 20 to 90 °C at the rate of 0.5 °C/min. Ellipticity was measured at 203 and 207 nm for GroES and gp31, respectively. Doubly averaged CD spectra were taken with 10° steps to control the state of the sample. For the thermal unfolding plots, the ellipticity values were normalized on the maximal value for each sample and plotted against the temperature. The transition temperatures were defined as the temperature value at which half of the analyte was unfolded.

### Mass Spectrometry

MS and tandem MS data were collected on the Thermo Scientific Orbitrap Exactive Plus mass spectrometer modified and optimized for transmission and detection of ions with *m*/*z* up to 50 kTh as described previously [37]. For ion isolation, we used a standard quadrupole mass filter from a Q-Exactive instrument with a modified electronic board featuring a decreased resonance frequency of 284 kHz enabling an upper mass-selection limit above 20 kTh [38, 39]. The samples were buffer exchanged into 10 mM ammonium acetate at pH 6.8 and diluted to a final concentration of 10 μM immediately before the experiment. All acquisitions were collected at the 64-ms transient times equivalent to 17,500 resolution at *m*/*z* 200. For each final spectrum, a minimum of 10 scans was combined, containing 10 μscans at 100 ms injection times. In all MS and IM-MS experiments, the samples were sprayed from gold-coated borosilicate glass needles produced in-house [40].

### Ion Mobility-Mass Spectrometry

IM-MS measurements were performed in positive ion mode using an electrospray ionization quadrupole ion mobility time-of-flight (ESI-Q-IM-TOF) instrument (Synapt HDMS, Waters, UK) equipped with a Z-spray nano-electrospray ionization source. To retain complexes intact in the gas phase and improve collisional cooling and transmission of ions, pressure in the source region was elevated to 8 mbar. Argon was used as a collisional gas in the trap region at a pressure of 3 × 10^−2^ mbar. The ion mobility cell was filled with nitrogen (7.5 × 10^−1^ mbar). Wave height of 12 and 15 V and wave velocity of 300 and 500 m/s were used for the analyzed proteins in heptameric and monomeric forms, respectively.

### Data Processing

The Orbitrap MS data were automatically processed using an in-house built software. The IM-MS data were manually extracted using DriftScope 3.0 (Waters, UK) and converted to text format using MSConvert utility from the ProteoWizard 3 suite [41]. Calculations of solvent accessible surface area (SASA) were performed by using GETAREA scripts from http://curie.utmb.edu/getarea.html. “Contact Surface” script was taken from https://pymolwiki.org/index.php/Contact_Surface. Number of H bonds were calculated with the PyMOL script *list_hbonds.py* from http://pldserver1.biochem.queensu.ca/~rlc/work/pymol/. All further data analysis was performed using R [42] and ggplot2 [43]. The drift time and collision cross-section heat map plots were produced using modified versions of the CIUSuite scripts [44].

## Results

### Distinct Gas-Phase Collision Induced Dissociation of the GroES and gp31 Heptamers

To initiate investigations on the gas-phase dissociation behavior of GroES and gp31, we first analyzed both complexes by native MS. GroES and gp31 were dissolved in aqueous ammonium acetate buffer adjusted to a pH ~ 6.8. Samples with a final concentration of the monomer of 10 μM were sprayed from the nano-electrospray (nano-ESI) ion source into Q-Exactive EMR Orbitrap mass spectrometer, modified as described earlier [37, 38]. The resulting native mass spectra of GroES and gp31 are shown in the Figure 1d, e. In the case of GroES, the majority is present in the form of heptamers, with only marginal quantities of the trimer and the tetramer (Figure 1d). Gp31 is present in a substantially higher variety of oligomeric forms (Figure 1e), which may hint at a potentially weaker stability of the gp31 heptamers. We conclude from these data that both GroES and gp31 are most stable as heptamers both in the solution and in the gas phase.

**Figure 2:**
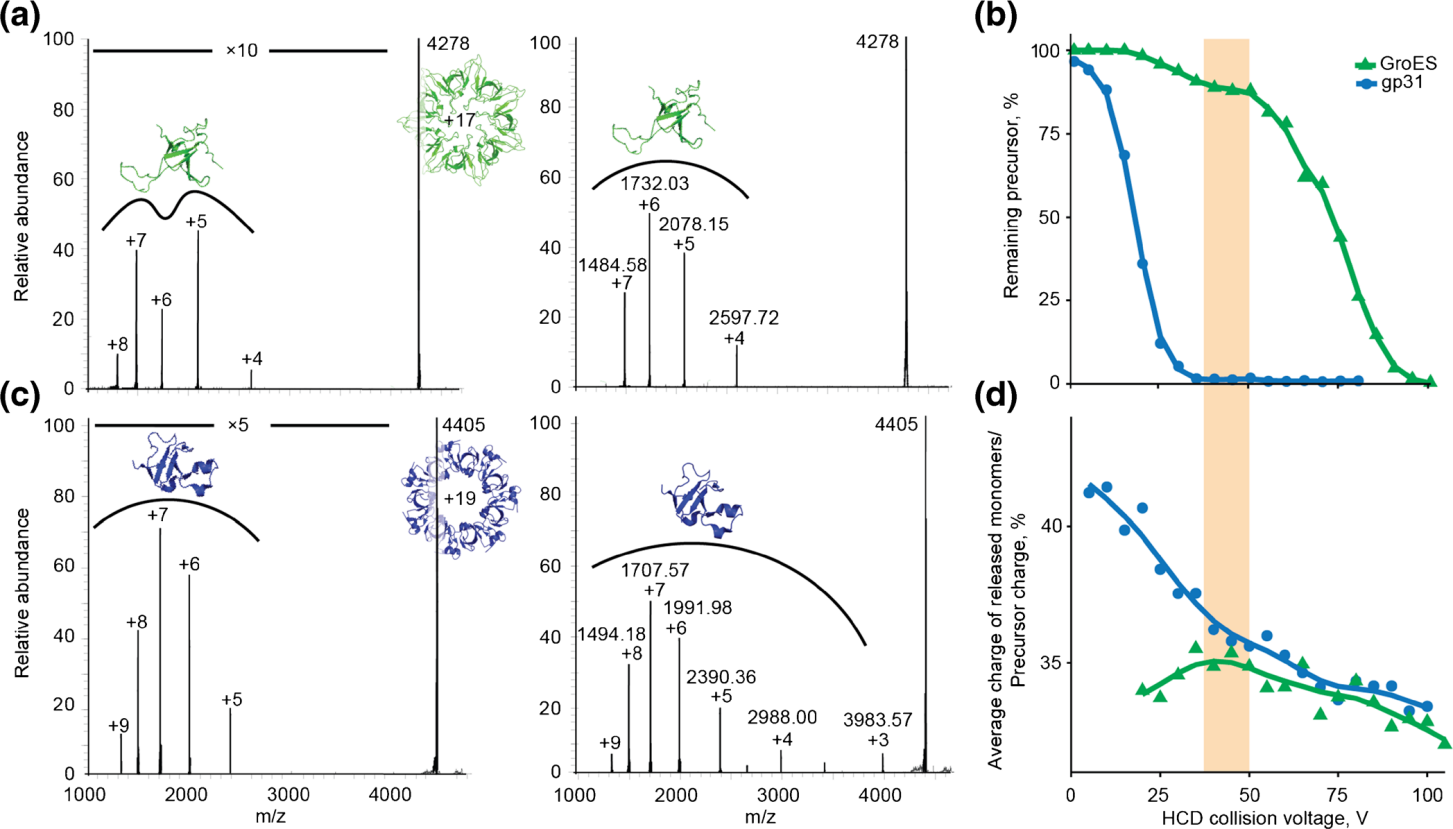
Tandem mass spectra and breakdown curves of mass-selected GroES and gp31 heptamers. Tandem MS of (**a**) GroES^17+^, HCD voltage 40 V (left) and HCD voltage 75 V (right), and (**c**) gp31^19+^, HCD voltage 20 V (left) and HCD voltage 25 V (right); × 5 and × 10 are magnification factors for indicated region. (**b**) Breakdown curves of GroES^17+^ and gp31^19+^ against range of applied collision voltages. (**d**) Average charge of monomers released from GroES^17+^ and gp31^19+^ as percentage of precursor charge plotted against the applied HCD voltages

To investigate gas-phase stability of the two heptamers, we used a high-mass quadrupole mass filter to isolate individual charge states of each complex ion and subjected them to collisional activation. It has been previously reported that, within a given charge-state envelope, the lower-charged ions resemble the proteins native state better than higher-charged ions [45]. Hence, we chose the lowest detectable, although still relatively abundant, charge states of GroES (+ 17) and gp31 (+ 19) for the comparison of their response to collisional activation (Figure 2a, c). The breakdown curve for each heptameric complex is plotted against the collision voltage, which was applied at the entrance of higher-energy collisional dissociation (HCD) cell (Figure 2b). In order to reduce bias introduced by comparison of different charge states we also produced breakdown curves for the same charge state (+ 19) of GroES and gp31 (Figure S1). Direct comparison of the two heptamers highlights differences in the gas-phase stability of GroES and gp31 upon collisional activation. First, GroES requires substantially higher voltages to dissociate than gp31. Considering that the MW of gp31 is about 20% higher than the MW of GroES, these results imply a significantly weaker inter-subunit interactions and/or lower stability of gp31 as compared to GroES. Second, the breakdown curve of the GroES follows a clear double sigmoidal shape, as opposed to a single sigmoidal curve obtained for gp31 (Figure 2b). The double sigmoidal breakdown curve suggests (at least) two co-existing dissociation pathways, whose relative contributions depend on the voltage and hence the kinetic energy of the ion. Alike data on the other charge states of GroES and gp31 (data not shown) corroborated these findings, wherein at low collisional energies gp31 consistently displayed unimodal distribution of released monomers, while GroES displayed relatively less abundant bimodal monomer distribution.

Tandem MS spectra can offer a greater level of detail about the dissociation process by allowing for a qualitative comparison of the dissociation spectra at varying energy. The tandem MS spectra taken before and after the first plateau of the GroES breakdown curve display strikingly different dissociation patterns. At low voltages, the distribution of dissociation product intensities adopts an atypical bimodal form, with extrema around charges + 7 and + 5 of the released monomers (Figure 2a; left). At the higher energies, the monomer intensity distribution is more unimodal (Figure 2a; right). Distinct monomer distributions at low collision energies might indicate the co-occurrence of two distinct dissociation mechanisms, with only one becoming prominent at higher collision energies. In contrast, intensities of the monomers released from gp31 follow a unimodal distribution throughout all applied collision voltages, pointing at a more facile and uniform dissociation pathway (Figure 2c).

The charge states of protein ions produced via electrospray are known to correlate with the degree of unfolding [46, 47], as more extended conformations offer more surface area to accommodate protons. Similarly, more unfolded subunits released from protein complexes upon collision-induced activation in the gas phase harbor more protons than subunits ejected with more compact structures [48]. Hence, the bimodal charge distributions hint at the presence of distinct unfolding states of the GroES monomers ejected from the complex upon collisional activation. This bimodal charge distribution of released monomers is not observed for gp31. To illustrate these differences, we plotted the average charge state of the monomers ejected from GroES and gp31 heptamers as fraction of precursor charge at different HCD collision voltages (Figure 2d).

At low HCD voltages, the normalized average charge of ejected monomers for GroES is lower than that for gp31, indicating that at low collision energies, the ejected subunits experience a relatively low degree of unfolding. With elevation of collision energies, distribution of released monomers for GroES starts to shift toward higher charge states, indicating that monomer unfolding prior to ejection starts to prevail. At a HCD voltage of ~ 40 V, there is a transition point, after which both GroES and gp31 display similar dissociation behavior. The transition point in the GroES curve coincides with the plateau of the breakdown curve (Figure 2b, d; highlighted with light orange), indicating that from this point on, the second dissociation pathway is taking over. Consistent decrease of normalized average charge of released monomers for gp31 (Figure 2d) agrees with the predicted uniform dissociation pathway.

### Distinct Collision Induced Unfolding of the GroES and gp31 Heptamers Revealed by IM-MS

**Figure 3:**
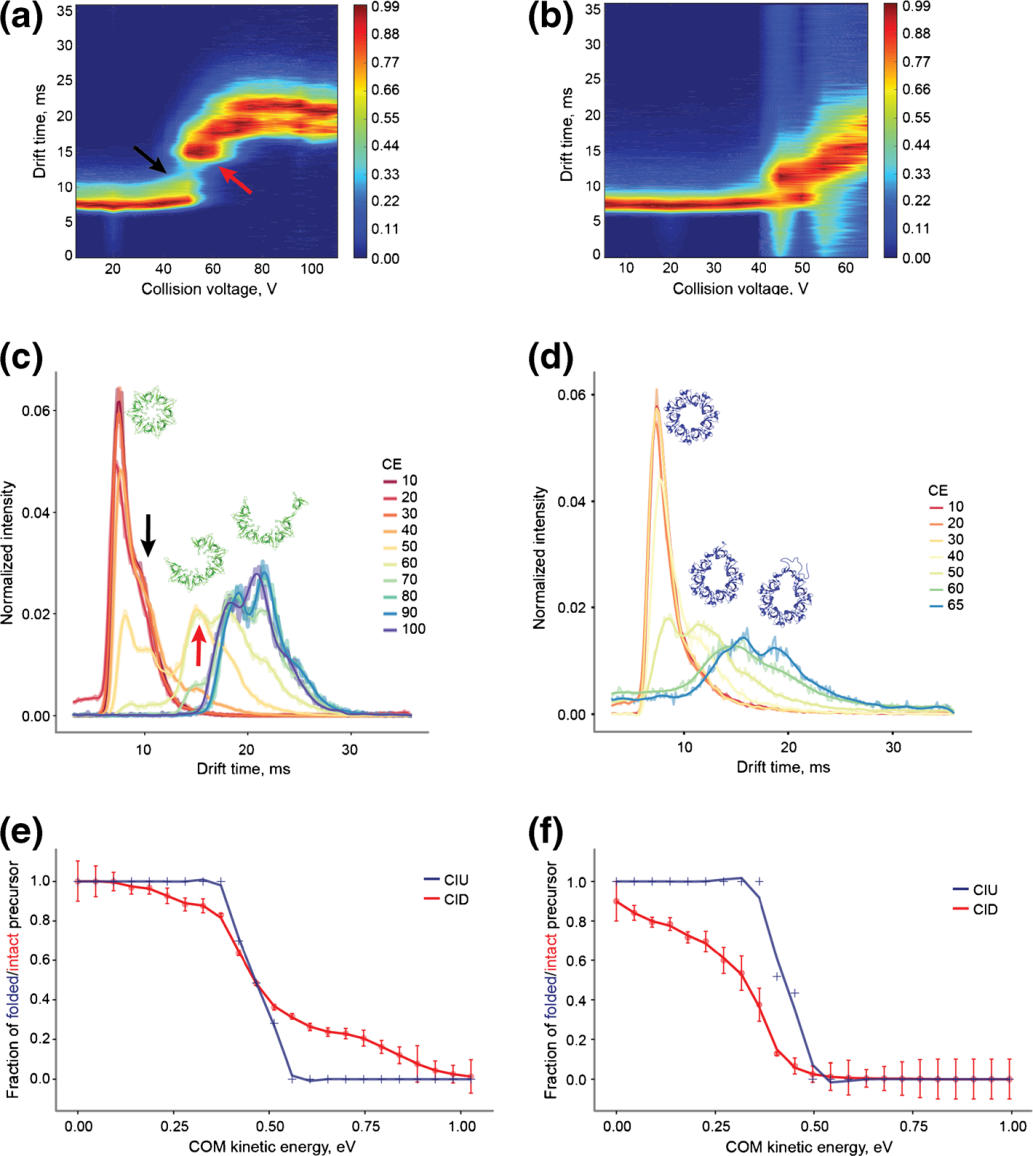
Collision induced dissociation (CID) and collision induced unfolding (CIU) characteristics of GroES and gp31 heptamers. (**a**) 2D heat map representing the unfolding of GroES^17+^. (**b**) 2D heat map representing the unfolding of gp31^19+^. (**c**) Arrival time distributions (ATD) of GroES^17+^ ions at various collision energies (CE). (**d**) ATDs of gp31^19+^ ions at different collision energies. (**e**) Comparison between the CIU and CID of GroES^17+^ ions. (**f**) Comparison between the CIU and CID of gp31^19+^ ions. Structures in (**c**) and (**d**) depict predicted unfolding events

To further investigate the interplay between dissociation and unfolding of heptameric GroES and gp31, we next performed ion mobility (IM-MS) experiments. First, we examined the conformational changes of the heptameric ring complexes upon collisional activation through collision induced unfolding (CIU) [44, 49]. As before, we chose the lowest detectable charge states of both GroES (+ 17) and gp31 (+ 19) also to enable a direct comparison between their behavior under CIU and CID conditions (Figure 3). Prior to IM-MS measurements, the ions were subjected to activation by collisions with an inert gas (Ar) at varying energies. The degree of unfolding was monitored by following the changes in ion drift times. Unfolding of a protein in the gas phase relates generally to an increase of its geometrical cross section [50], resulting in a shift in arrival time distribution (ATD) in IM-MS.

The 2D heat maps of GroES and gp31 display several notable differences (Figure 3a, b). Unfolding of GroES is mainly represented with a sharp shift of its ATD (Figure 3a, c; red arrow), followed by less dramatic subsequent second and third unfolding events. Prior to the first major shift of the ATD, the GroES heptamer displays a broadening of the driftogram (Figure 3c; black arrow). This change at low collision energy is accompanied by released monomers with relatively low average charge (Figure 2d), which hints at their partly retained folded state. At this point, precursor dissociation occurs to a small extent probably due to strong inter-subunit interactions. The sharp shift of ATD for the GroES heptamer is likely associated with disruption of the inter-subunit interface and opening of the heptameric ring (Figure 3a, c; red arrow). At higher collision voltages, the GroES heptamers are present in two co-existing relatively abundant extended conformations. These smaller conformational changes can be attributed to unfolding of the terminal monomers of the resulting extended structure, prior to their elimination from the heptamer.

In contrast, the gp31 heptamer does not display any sharp shifts in ATD upon activation. Elevation of collision voltages leads to gradual increase of the drift time and significant broadening of the ATD (Figure 3b, d). This behavior can be best explained by gradual unfolding of one or several subunits that are still forming a heptameric ring. We detected all the discussed IM-MS features also for the other relatively abundant charge states of the GroES and gp31 heptamers (Figure S2).

The distinct behavior of the two heptamers upon both CID and CIU becomes clearer upon plotting their unfolding curves (percentage of folded heptamer) along with the breakdown curves (percentage of intact precursor) plotted against the center-of-mass energy of the ion (*E*_*kin*_^*COM*^) (Figure 3e, f). In case of GroES, there are again two separate regions: the low energy region (*E*_*kin*_^*COM*^ < 0.5 eV) and the higher energy region (*E*_*kin*_^*COM*^ > 0.5 eV) (Figure 3e). At the lower energies, the majority of the heptamers have mostly still retained their original compact conformation. All deposited energy at this point goes into disruption of the inter-subunit interfaces, leading to an ejection of relatively folded monomers. However, this occurs only to a fraction of the heptameric precursor. At higher energies, the heptamer adopts a more extended conformation, which likely happens prior to dissociation and is associated with unfolding of the GroES subunit after disruption of the ring structure. The dissociation products in this energy regime are expected to be more unfolded. Interplay of these two dissociating mechanisms for GroES is reflected in the bimodal monomer charge distribution observed at 40 V collision energy (Figure 2a; left).

Despite our efforts to preserve the intact state of gp31 heptamer, we still observed a certain degree of dissociation even at the lowest energy (Figure 3f). However, the narrow ATD of the precursor heptamer (Figure 3d) assured that the remaining part of the heptameric precursor is still largely in the original intact native-like form. This observation could be explained by relatively weak inter-subunit interfaces, which are likely to be disrupted simultaneously. Interestingly, the high average charge of the released monomers (> 40% of precursor charge) at low collision energy (Figure 2d) indicates that this energy is not only enough to disrupt the binding of the subunit within the complex, but additionally is sufficient to unfold the subunit prior to ejection. With the increase of energy, the precursor dissociates further, while the remaining intact precursor retains a compact state. Broadening of the ATD with elevation of collisional energy is accompanied by significant decrease of the overall precursor intensity. We hypothesize that at this point in parallel with disruption of binding interfaces and unfolding of the ejected single subunits, multiple subunits might undergo unfolding competing for charges, as recently proposed by in silico simulations for tetrameric complexes [48]. More stochastic ejection of variously unfolded subunits, rather than release of the most unfolded monomer, would explain presence of low charged monomers down to 3 + (~ 15% of precursor charge state) along with higher-charged monomers at high collision energy (Figure 2c; right).

### Stability of GroES and gp31 in the Solution

**Figure 4:**
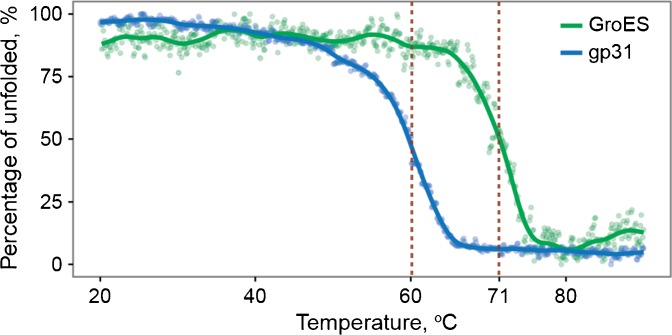
Thermal unfolding curves of the GroES and gp31 heptamers in the solution, as monitored by changes in the circular dichroism (CD) spectra. The extracted melting transitions are 71 and 60 °C, respectively, for GroES and gp31

The striking behavior we observed in the gas phase, whereby GroES heptamers dissociate at much more elevated activation energies than the structural homologue gp31 was somewhat unanticipated. Therefore, we set out to test the stability of these two heptamers in the solution. We performed a thermal unfolding assay by recording circular dichroism (CD) spectra at variable temperatures. Upon heating, both GroES and gp31 assemblies experience clear unfolding transitions (Figure 4). Additionally, we used the first derivative of the ellipticity versus the temperature to confirm the transition point where a maximum rate of the ellipticity change is observed (Figure S3). For both assemblies, the transition represented a sharp increase of the unstructured content. In these assays in the solution, GroES displayed a significantly higher stability, undergoing a sharp unfolding transition at 71 °C (in accordance with published data [51]), whereas for gp31, the unfolding transition point was observed at 60 °C (Figure 4; dashed lines). This strongly suggests that also in the solution, more energy is required to unfold GroES than gp31. Seemingly, this order of stability in the solution is retained in the gas phase. Next, we sought to further explain this behavior inspecting the inter-subunit interfaces within the GroES and gp31 heptamers.

### Inter-Subunit Binding Is More Stabilized in GroES than in gp31

It has previously been argued that the chemical nature of the binding interface (solvent accessible surface area, number of salt bridges, amount and strength of hydrogen bonding events, etc.) can be correlated to the dissociation behavior of protein complexes upon activation in the gas phase [24, 52, 53]. Zooming in on the binding interfaces of GroES and gp31, using the available crystal structures, revealed several differences (Table 1). The contact area between the subunits was calculated using the Contact Surface script, that calculates the SASA buried in the interface between the interacting molecules. The contact areas within the GroES heptamer are 7% larger than in gp31. Considering that GroES is about 15% smaller than gp31, this provides a strong indication of tighter inter-subunit interactions within GroES. Next, we determined the number of inter-subunit hydrogen bonds, using a distance cutoff and bond angle restriction of 3.2 Å and 55°, respectively. Again, we observed that the GroES heptamer is stabilized by more hydrogen bonds than gp31 (Table 1). As in both assemblies, we identified only one inter-subunit salt bridge, in both cases between an arginine and a glutamic acid, we argue that this does not contribute to the difference. Overall, our analysis of the interaction surface between monomers within the GroES and gp31 heptamers indicates that it is more stabilized in GroES, when compared to gp31, likely explaining the higher melting temperature observed for GroES, and the higher resistance to gas-phase dissociation, when compared to gp31.

**Table 1:** Summary of the Characteristics of the Contact Areas Between the Subunits in Heptameric GroES and gp31 Extracted from the Available Crystal Structures (PDB 1AON (GroES), 1G31 (gp31))

	Contact area (Å^2^)	H bonds	H bond distance (Å)	Salt bridges
GroES	813 ± 11	7 ± 1	2.7 ± 0.2	R37-E76
gp31	754 ± 8	6 ± 1	2.8 ± 0.2	R77-E44

### GroES and gp31 Unfold and Dissociate in the Gas Phase Via Distinct Mechanisms

Based on the experimental data, we propose the following model to describe the distinctive gas-phase behavior of GroES and gp31. When the internal energy of the ionized heptameric complexes increases because of collisions with the buffer gas, its inter-subunit and intra-subunit noncovalent interactions begin to weaken. For most protein assemblies reported to date, this process would lead to the specific unfolding of one of the subunits, followed by its elimination from the assembly [54], with unfolding typically happening at lower energies than dissociation [55]. Distinctively, GroES heptamer dissociation seems to proceed through a combination of two mechanisms. The first mechanism involves the partial unfolding of an individual subunit that leads to disruption from neighboring inter-subunit interfaces and dissociation of the monomer. The partial unfolding can be seen by the shoulder being formed at the right edge of the ATD of GroES (Figure 3c; black arrow). This mechanism is prevalent at lower activation energies. As the activation energy increases, the second mechanism starts to kick in and take over. More energetic collisions begin to destabilize inter-subunit interfaces which leads to disruption of the ring. Further redistribution of energy likely causes destabilization and unfolding of two terminal subunits of the resulting extended structure, which is reflected in two minor ATD shifts that happen directly after the first major shift (Figure 3c). Finally, part of the resulting extended heptamers dissociate into a monomer and a hexamer, completing the second dissociation mechanism. The GroES monomers, produced via different dissociation mechanisms, become unfolded to a different degree, which explains the bimodal charge distribution of the released monomer in the low collisional energy regime (Figure 2a; left). Both the breakdown curves (Figure 2b; green) and the combined CIU/CID plots (Figure 3e) suggest that there are two energy zones that can be roughly separated by the point where the precursor ATD undergoes a sharp shift (at *E*_*kin*_^*COM*^ ~ 0.48 eV, CID voltage ~ 50 V). Considering the previous explanation, it is likely that the first mechanism is prevalent at the lower energies and the second—in the high-energy regime.

Gp31, in contrast, does not change its dissociation behavior over the entire activation energy range. Due to the intrinsic weaker inter-subunit interface and less stable tertiary structure, even a marginal increase of energy leads to destabilization of the fold of individual subunits and facile elimination of a monomer. With increase of collisional energy, more than one subunit undergoes unfolding with subsequent release from the complex. That likely explains why the monomeric dissociation products are unfolded to a various degree, which is manifested in a wide distribution of the monomer charge states at elevated collision voltages (Figure 2c; right).

## Conclusion

The GroES and gp31 protein heptamers are functional and structural homologues. Both protein complexes act as molecular co-chaperonins partnering with the tetradecamer GroEL to assist in the folding of nonnative polypeptide chains in *E. coli*. Here, we first observed that in the gas phase, the GroES heptamers are strikingly more stable than the gp31 heptamers, resulting in very distinctive breakdown curves as obtained upon collisional activation as well as distinctive collision induced unfolding patterns, which we determined by IM-MS. Subsequently, we probed the stability of GroES and gp31 heptameric complexes in the solution using thermal unfolding assays. GroES showed a sharp melting/unfolding transition at 71 °C, whereas for gp31, this transition occurs at 60 °C. Conclusively, gp31 is less stable than GroES both in the gas phase and in the solution. Analyzing the available high-resolution structures of the GroES and gp31 heptamers, we could deduce that the subunit interfaces within GroES are larger and harbor more hydrogen bonds comparatively to gp31. These findings underscore the higher stability of GroES when compared to gp31 in the solution. Overall, our data reveal that although the GroES and gp31 heptamers are functionally and structurally homologous, they exhibit striking differences in stability and unfolding. Finally, we conclude from our data that the solution phase structural properties of GroES and Gp31 complexes are partially retained upon transfer into the gas phase, which makes mass spectrometry-based approaches useful for complementing the solution-phase analysis of protein complex stabilities.

## Electronic Supplementary Material

ESM 1 (DOCX 1393kb)
