# Supplementary material for: Distinct Stabilities of the Structurally Homologous Heptameric Co-Chaperonins GroES and gp31
Source: J Am Soc Mass Spectrom. 2018 May 7;30(1):7–15. doi: 10.1007/s13361-018-1910-5 (PMC6318259; doi:10.1007/s13361-018-1910-5)

**Supporting Information**

**Distinct stabilities of the structurally homologous**

**heptameric co-chaperonins GroES and gp31**

Andrey Dyachenko^1,2^, Sem Tamara^1,2^, Albert J. R. Heck^1,2,*^

^1^ Biomolecular Mass Spectrometry and Proteomics, Bijvoet Center for Biomolecular Research and Utrecht Institute for Pharmaceutical Sciences, Utrecht University, Padualaan 8, 3584 CH Utrecht, The Netherlands

^2^ Netherlands Proteomics Centre, Padualaan 8, 3584CH Utrecht, the Netherlands

^*^ To whom correspondence should be addressed: [a.j.r.heck@uu.nl](mailto:a.j.r.heck@uu.nl)

Contribution to Special Focus issue honoring Carol V. Robinson’s election to the National Academy of Sciences, USA.

Figure S1. Breakdown curves of 19+ charge state of heptameric GroES and gp31.


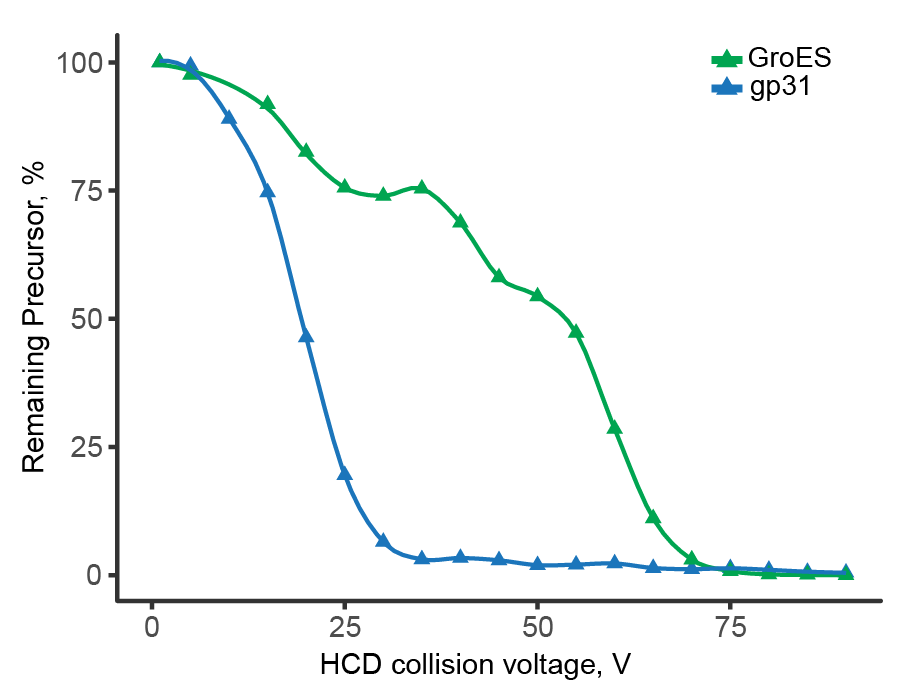


Figure S2. IM-MS 2D heatmaps for several charge states of GroES and gp31.


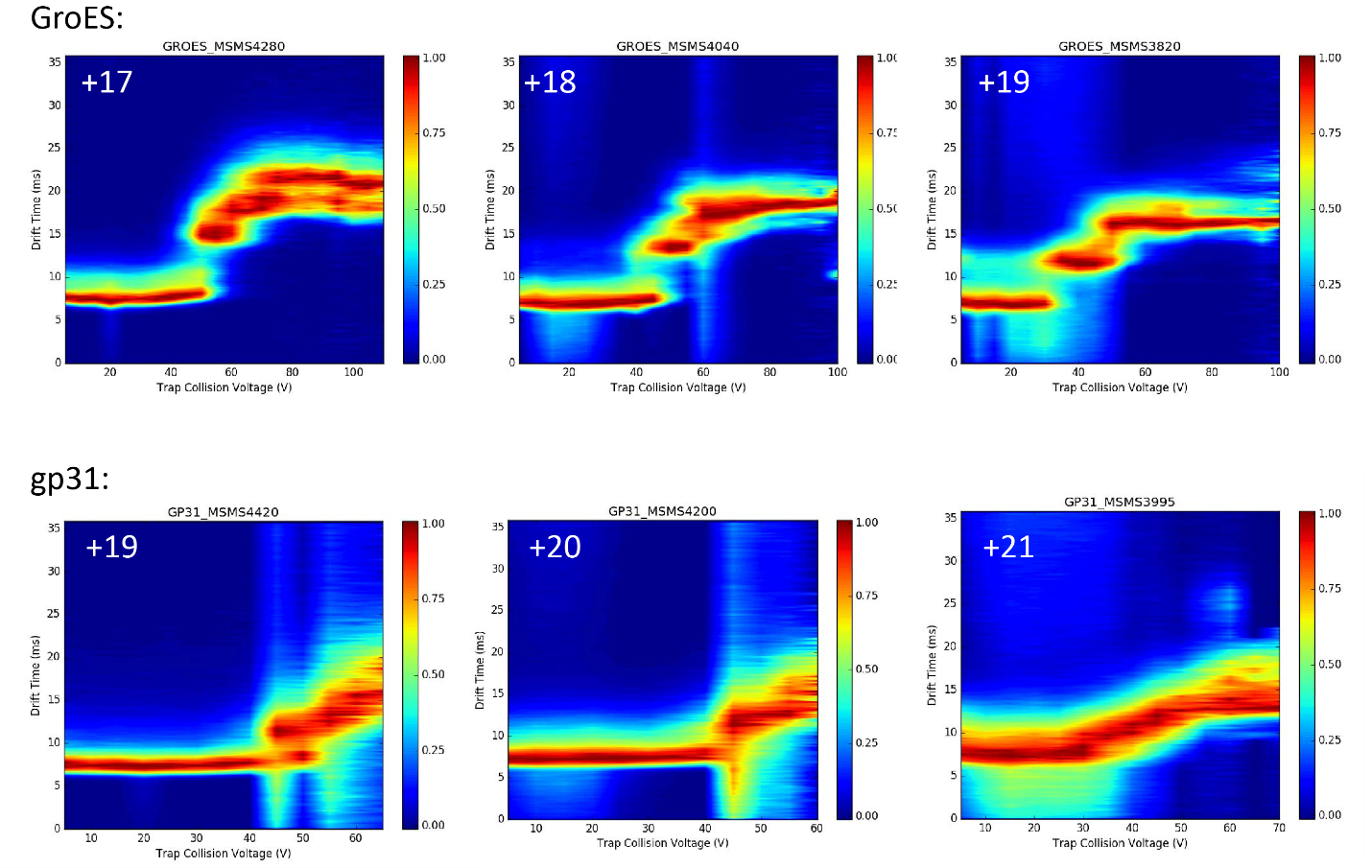


Figure S3. First derivative of the molar ellipticity of GroES and gp31 plotted versus the temperature.


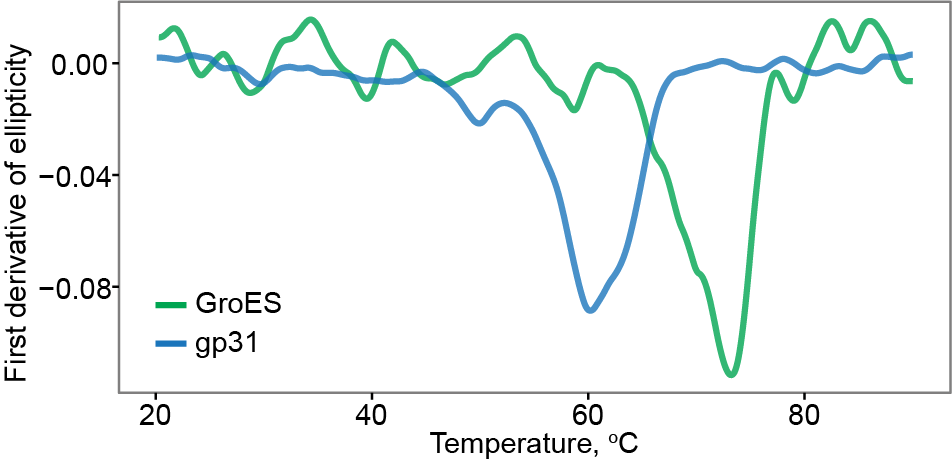

Supplement: Supplementary file 1 — (DOCX 1393kb) [file 13361_2018_1910_MOESM1_ESM.docx]
